# Columnar distribution of activity dependent gabaergic depolarization in sensorimotor cortical neurons

**DOI:** 10.1186/1756-6606-5-33

**Published:** 2012-09-24

**Authors:** Jaekwang Lee, Junsung Woo, Oleg V Favorov, Mark Tommerdahl, C Justin Lee, Barry L Whitsel

**Affiliations:** 1 Departments of Biomedical Engineering, University of North Carolina at Chapel Hill, CB#7575, Chapel Hill, NC, USA; 2 Department of Cell and Molecular Physiology, University of North Carolina at Chapel Hill, CB#7575, Chapel Hill, NC, USA; 3 WCI Center for Functional Connectomics, Institute of Science and Technology (KIST), Seoul, 136-791, Korea

## Abstract

**Background:**

GABA, the major inhibitory neurotransmitter in CNS, has been demonstrated to paradoxically produce excitation even in mature brain. However activity-dependent form of GABA excitation in cortical neurons has not been observed. Here we report that after an intense electrical stimulation adult cortical neurons displayed a transient GABA excitation that lasted for about 30s.

**Results:**

Whole-cell patch recordings were performed to evaluate the effects of briefly applied GABA on pyramidal neurons in adult rodent sensorimotor cortical slice before and after 1 s, 20 Hz suprathreshold electrical stimulation of the junction between layer 6 and the underlying white matter (L6/WM stimulation). Immediately after L6/WM stimulation, GABA puffs produced neuronal depolarization in the center of the column-shaped region. However, both prior to or 30s after stimulation GABA puffs produced hyperpolarization of neurons. 2-photon imaging in neurons infected with adenovirus carrying a chloride sensor Clomeleon revealed that GABA induced depolarization is due to an increase in [Cl^-^]_i_ after stimulation. To reveal the spatial extent of excitatory action of GABA, isoguvacine, a GABA_A_ receptors agonist, was applied right after stimulation while monitoring the intracellular Ca^2+^ concentration in pyramidal neurons. Isoguvacine induced an increase in [Ca^2+^]_i_ in pyramidal neurons especially in the center of the column but not in the peripheral regions of the column. The global pattern of the Ca^2+^ signal showed a column-shaped distribution along the stimulation site.

**Conclusion:**

These results demonstrate that the well-known inhibitory transmitter GABA rapidly switches from hyperpolarization to depolarization upon synaptic activity in adult somatosensory cortical neurons.

## Background

It is widely accepted that the principal mechanism for triggering neuronal hyperpolarization/inhibition in adult neocortex is synaptic transmission involving GABA action via GABA_A_ receptors [1-5]. Recently reported single-neuron recording observations, however, raise the intriguing possibility that, similar to its bipolar (+/−) activity-dependent action on adult hippocampal neurons [6-13], the synaptic influence of GABA on neurons in primary somatosensory cortex (SI) in adult subjects may not be restricted to hyperpolarization – i.e., either an ongoing or very recent exposure of an SI neuron to a suprathreshold mechanical skin stimulus may be accompanied by conversion of the GABA_A_ receptor-mediated effect of GABA on that neuron from hyperpolarization/inhibition to depolarization/excitation [14].

The principal goal of the series of *in vitro* experiments reported in this paper was to determine if, to what extent, and for what period of time the synaptic action of GABA on pyramidal neurons in the adult rodent sensorimotor cortical slice is altered subsequent to exposure to a brief (1 s) episode of repetitive (20 Hz) extrinsic excitatory afferent drive. The observations (described previously in abstract format [15]) reveal that the response of the sensorimotor cortical slice to a 1 s of suprathreshold 20 Hz stimulation of a discrete site at the layer 6–white matter (L6/WM) junction restricted on columnar space as a consequence of the activity-dependent, bipolar (+/−) alteration of pyramidal neuron membrane potential associated with GABA action on GABA_A_ receptors. The alteration of slice’s spatially distributed response to extrinsic excitatory afferent drive persists for more than 20s after stimulus termination. Potential implications for the SI encoding/representation of environmental events of the activity-dependent, bipolar action of GABA revealed by the experiments of this study are identified and discussed.

## Results

### Assessment of the neuronal action of GABA

**Figure 1:**
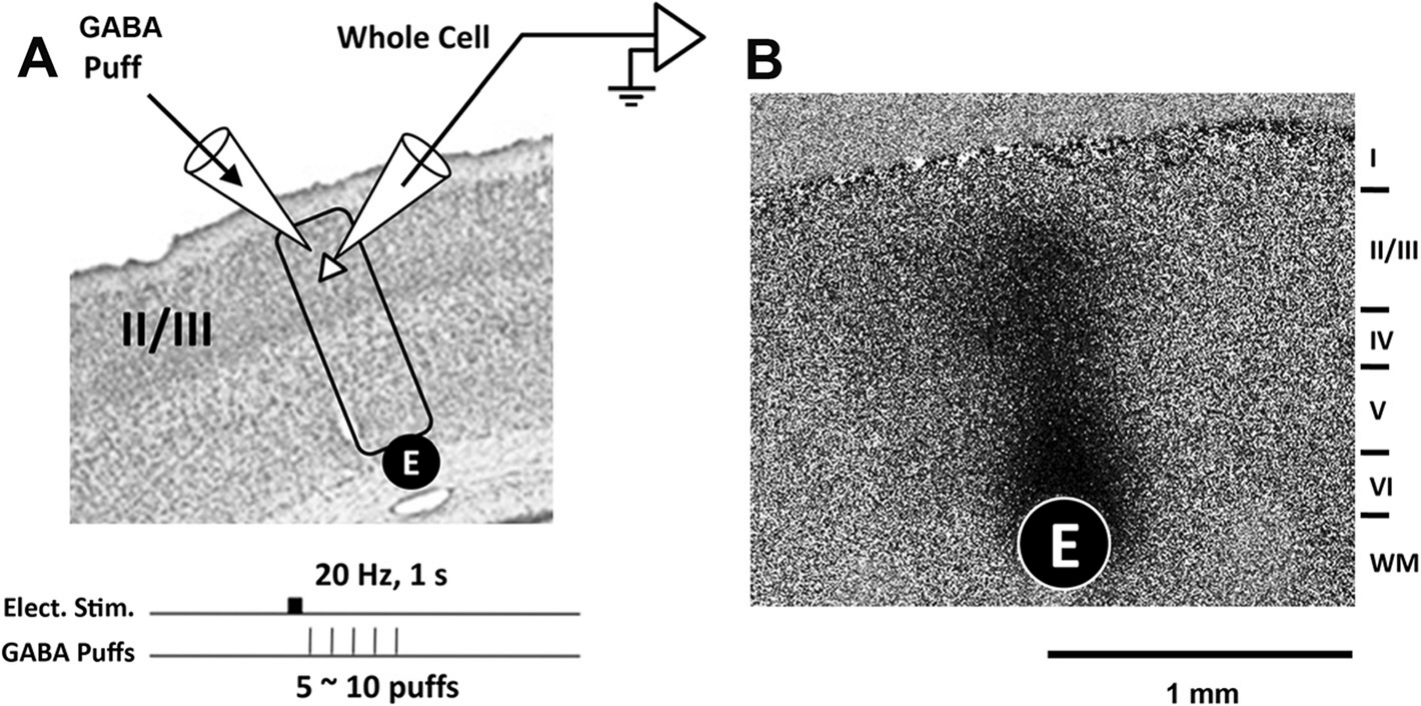
**Experimental design.A**: Diagram showing experimental setup and protocol. Filled circle labeled “E” is a site at which a bipolar stimulating electrode is placed into contact with the layer 6/white matter junction. Rectangle encloses the cortical column-shaped region activated most vigorously by repetitive electrical stimulation applied via the electrode at E. Open triangle depicts an upper layer pyramidal neuron whose activity is recorded with whole-cell patch clamp recording electrode and amplifier. Micropipette filled with GABA is shown at top left with its tip in an extracellular position in close proximity to the recorded pyramidal neuron – a position enabling pressure-ejection (“puff”/local extracellular release) of GABA at selected times subsequent to the application of repetitive excitatory afferent drive at the L6/WM site contacted by the stimulating electrode. **B**: Representative example of column-shaped OIS response to the L6/WM stimulus (20-200 μA pulses delivered at 20 Hz).

Each L6/WM stimulus (delivered via a cylindrical bipolar electrode; outer diameter 250 μm) was 1 s in duration, consisted of a 20-pulse train of constant-current pulses, and was applied at the L6/WM junction at a site located centrally in the slice (Figure 1A). The OIS imaging method enables visualization of the spatially restricted region in the sensorimotor cortical slice in which pyramidal neuron spike discharge activity remains elevated throughout the entire 1 s period of the 20 Hz L6/WM stimulation. Although the pyramidal neurons in a spatially extensive region of the slice (typically ≥4-5 mm; Lee et al. 1992) are activated by a single-pulse, suprathreshold L6/WM stimulus, the region that develops an OIS response to 1 s, 20 Hz L6/WM stimulus consistently is: (i) columnar in shape (~300 μm in diameter); (ii) located immediately above the stimulus site; and (iii) extends continuously across layers 2–6 (Figure 1B).

In the study of each slice, a layer 2–3 pyramidal neuron located within the column-shaped region that developed an OIS in response to the L6/WM stimulus was selected, and after stable whole-cell patch recordings were obtained, the tip of a GABA-containing micropipette was placed within 50-100 μm of the soma (using a micrometer and 3-axis positioner; NMN-25, Narishige Group Inc., Tokyo, Japan). This positional arrangement of the patch recording electrode and the GABA-containing micropipette enabled evaluation (by the application of positive air pressure to the GABA-filled micropipette; Picosprizter, 10-30 psi, 10-30 ms) of the effect of extracellularly-applied GABA on the neuron’s membrane potential prior to (“control” observations) and at a series of times following each L6/WM stimulus (“test” observations). For each neuron studied in this manner GABA was applied (“puffed”) 5–10 times, and in every case the response to at least 3 GABA applications/puffs was recorded prior to L6/WM stimulation. A 5 s interval separated successive GABA puffs delivered before or following the L6/WM stimulus.

### Synaptic action of GABA alters in response to stimulus-evoked excitatory afferent drive

**Figure 2:**
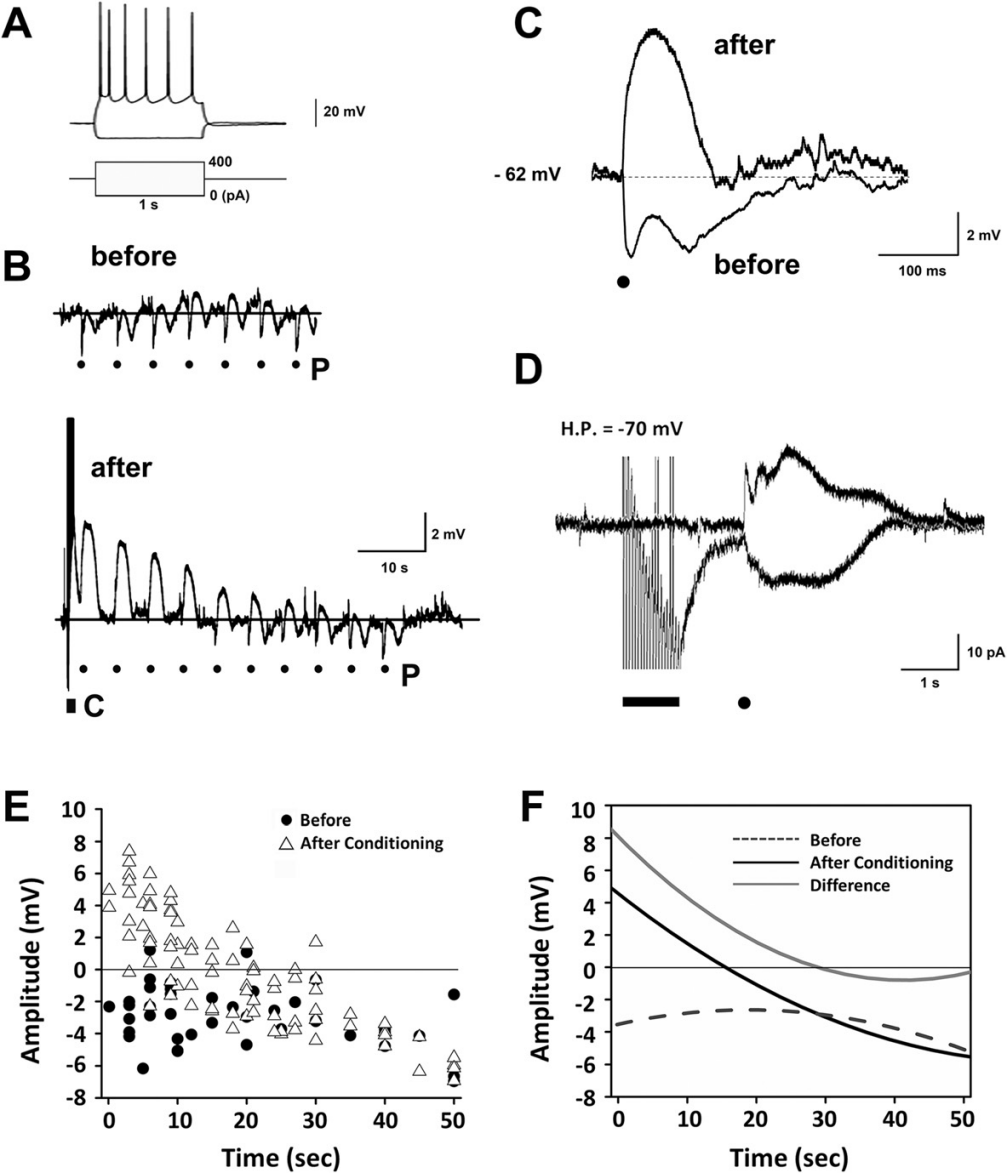
**GABA’s effect on exemplary upper layer pyramidal neuron prior to and following L6/WM stimulation.A**: Spike firing of the neuron during passage of transmembrane current – note that inward current elicits spike firing adaptation characteristic of the RS (regularspiking) class of cortical neurons. **B**: Upper and lower panels show membrane potential alteration induced by each puffer application of GABA before (upper trace) vs. after (lower trace) exposure to the 1 s 20 Hz L6/WM stimulus. Dots beneath each trace – denoted by P – indicate time of each of the GABA puffs applied before (upper trace) and after (lower trace) L6/WM stimulation. Filled rectangle (labeled C) indicates time of L6/WM stimulation. **C**: Time-course of depolarizing membrane potential response to the first GABA puff after electrical stimulation vs. the hyperpolarizing time-course of GABA puff response before L6/WM stimulation (average over 7 puffs shown in panel B). **D**: Voltage-clamp recordings showing GABA-evoked transmembrane currents prior to (top trace) and immediately following (bottom trace) L6/WM stimulation. Horizontal bar and dot at bottom indicate period of L6/WM stimulation and GABA puff, respectively. **E**: Amplitude of the response of each neuron to GABA is plotted as a function of time of each puff in the series of puffs delivered prior to (filled circles) and after (open triangles) L6/WM stimulation. **F**: Second-order least-squares approximations of the distributions of the GABA responses shown in panel A. Plot indicated by interrupted line shows 2nd order least squares approximation of observations obtained prior to layer VI stimulation (“Before”); plot indicated by bold solid line shows 2nd order least squares approximation of observations obtained after L6/WM stimulation (“After conditioning”). Light solid line labeled “Difference” is the difference between the “Before” and “After” distributions and estimates the time-course of the effect of L6/WM stimulation on the action of GABA.

The neurons selected for study were confined to layers 2–3 within the column-shaped region of the slice that: (i)developed an Optical Intrinsic Signal (OIS) in response to the L6/WM stimulus; and (ii)exhibited spike frequency adaptation properties characteristic of regular-spiking (RS) cortical neurons (Figure 2A). In this experiment the tip of the GABA-containing micropipette was positioned ~70 μm from the soma of an RS neuron residing in the center of the column-shaped territory activated by an L6/WM stimulus, membrane potential was recorded in the current-clamp mode, and GABA was applied extracellularly (“puffed”) in the immediate vicinity of the neuron both prior to and following a 1 s, 20 Hz suprathreshold electrical stimulus to the L6/WM stimulus site.

Figure 2B (trace at top labeled “before”) shows the membrane potential response of this exemplary neuron to each of the 7 GABA puffs delivered prior to L6/WM stimulation (dots under membrane potential trace in Figure 2B indicate onset of each puff). Each of these 7 GABA applications triggered a prominent hyperpolarizing membrane potential alteration whose magnitude and time course was relatively consistent from one application to the next. The average across-trial (n = 7) GABA-induced alteration of this neuron’s membrane potential before L6/WM stimulation is shown in Figure 2C (bottom trace labeled “before”). Clearly evident are the (i) extended duration, (ii) biphasic form (rapid onset of hyperpolarization and partial recovery, followed by a second, more protracted hyperpolarization and recovery), and (iii) high reproducibility of the GABA-evoked alteration of this neuron’s membrane potential prior to the exposure to L6/WM stimulation – attributes which correspond closely to published descriptions of the effect of GABA on cortical neuron membrane potential in the absence of ongoing or recent spike discharge activity [8,16].

In striking contrast, subsequent to the 1 s, 20 Hz L6/WM stimulus, GABA puff no longer was associated with hyperpolarization of this same neuron. Instead, for ~15 s subsequent to L6/WM stimulation this neuron’s response to each application of GABA was an unambiguous membrane potential depolarization. In addition, the observations obtained from this same neuron reveal that: (i)the amplitude of the GABA-evoked depolarization recorded subsequent to the L6/WM stimulus declined progressively with each successive puff in the series, (ii)only ~20-25 s after termination of the L6/WM stimulus did the response to GABA re-approximate both the polarity and amplitude of the GABA-mediated membrane potential alteration detected prior to the exposure to L6/WM stimulation.

As the final step in our study of the above-described exemplary upper layer RS neuron, the effect of GABA puff was re-determined with the amplifier in the voltage-clamp mode and the neuron’s membrane potential held at -70 mV. As Figure 2C reveals, whereas the transmembrane current flow triggered by GABA prior to delivery of the 1 s, 20 Hz L6/WM stimulus was outwardly directed (top trace), for ~15 s following the L6/WM stimulus the GABA-induced current flow was in the opposite, inward, direction (bottom trace; dot below traces in Figure 2D indicates GABA puff onset time).

The response to GABA puffs was determined for 16 upper layer pyramidal neurons using the same approach used to study the above-described exemplary neuron. The data points in Figure 2E show the amplitude of the membrane potential alteration evoked by GABA puff in each of these 16 neurons at the indicated time before (dots) vs. after (open triangles) the 1 s 20 Hz L6/WM stimulus. Whereas GABA puff before the exposure to L6/WM stimulation usually was accompanied by a modest (~2-4 mV) membrane potential hyperpolarization, for ~15 s following such stimulation the GABA-evoked membrane potential alteration was, in most instances, depolarizing. In addition, the data plot in Figure 2E reveals that although GABA puffs were associated with a relatively consistent alteration (hyperpolarization) of pyramidal neuron membrane potential at all times prior to L6/WM stimulation, during the 20-25 s interval that followed L6/WM stimulation the GABA-triggered alteration of membrane potential varied substantially and characteristically with increasing time – i.e., although GABA application routinely evoked a large-amplitude depolarization immediately subsequent to the L6/WM stimulus, each subsequent application resulted in a progressively smaller depolarization until at ~15-25 s after termination of the L6/WM stimulus the neurons’ response to GABA puff once again reverted to hyperpolarization.

A 3-step analytical approach was used to quantitatively estimate the time-course of the effect of L6/WM stimulation on the sensorimotor cortical pyramidal neuron response to extracellularly-applied GABA. First, the distribution of the responses of each neuron in the above-described sample of 16 upper layer pyramidal neurons to GABA before L6/WM stimulation was fitted by a least-squares 2nd order polynomial (dashed line plot labeled “Before” in Figure 2F). Second, the same data-fitting procedure was applied to the distribution of the post-L6/WM stimulation response of the same neuron sample to GABA (plot labeled “After Conditioning” in Figure 2F). And finally (step 3), the estimated time-course of the alteration of the effect of GABA following the L6/WM stimulus was obtained by subtracting the “Before” and “After Conditioning” 2nd order polynomials (the “Difference” plot in Figure 2F).

The outcome of the above-described analysis indicates that for a substantial post-stimulus period the action of puffer-applied GABA on the upper layer pyramidal neurons evaluated in the Series 1 experiments was depolarizing (the X-intercept of the “After Conditioning” plot in Figure 2F is at 16 s). In addition, the “Difference” plot in Figure 2F provides quantitative support for the authors’ interpretation that throughout this post-L6/WM stimulation period the magnitude of the GABA-evoked depolarization declined progressively. The X-intercept of the “Difference” plot in Figure 2F indicates that the effect of L6/WM stimulation on GABA action lasts for as long as 28 s.

Considered collectively, the observations in Figures 2 indicate that under the conditions the effect of GABA on upper layer pyramidal neurons is ***not fixed, but undergoes substantial, albeit transient, alteration subsequent to an exposure to vigorous excitatory afferent drive***. More specifically, the synaptic action of GABA on the upper layer pyramidal neurons located in the column-shaped territory of the slice immediately above the L6/WM stimulus site (the territory that develops a prominent OIS in response to that stimulation) is “bipolar and activity dependent” (i.e., it is depolarizing for ~15 s following a suprathreshold 1 s, 20 Hz L6/WM stimulus, but hyperpolarizing at all other times).

### Mechanism(s) underlying the bipolar, activity-dependent action of GABA on pyramidal neurons in the sensorimotor cortical slice

A substantial body of experimental findings reported previously by others has established that a co-occurrence of vigorous spike firing and GABA action on GABA_A_ receptors is accompanied by a prominent increase of [Cl^-^_i_ in the affected neurons [6,7,9,12,17-19]. This information led the authors to predict a priori that the vigorous pyramidal neuron activation that occurs in the column-shaped region of the rodent sensorimotor cortical slice located immediately above the L6/WM stimulus [20-22] is accompanied by an elevation of pyramidal neuron [Cl^-^_i_, which not only diminishes the hyperpolarization normally associated with GABA action on GABA_A_ receptors but, if the elevation of [Cl^-^_i_ is sufficiently large, converts these neurons’ response to GABA from hyperpolarization to depolarization.

**Figure 3:**
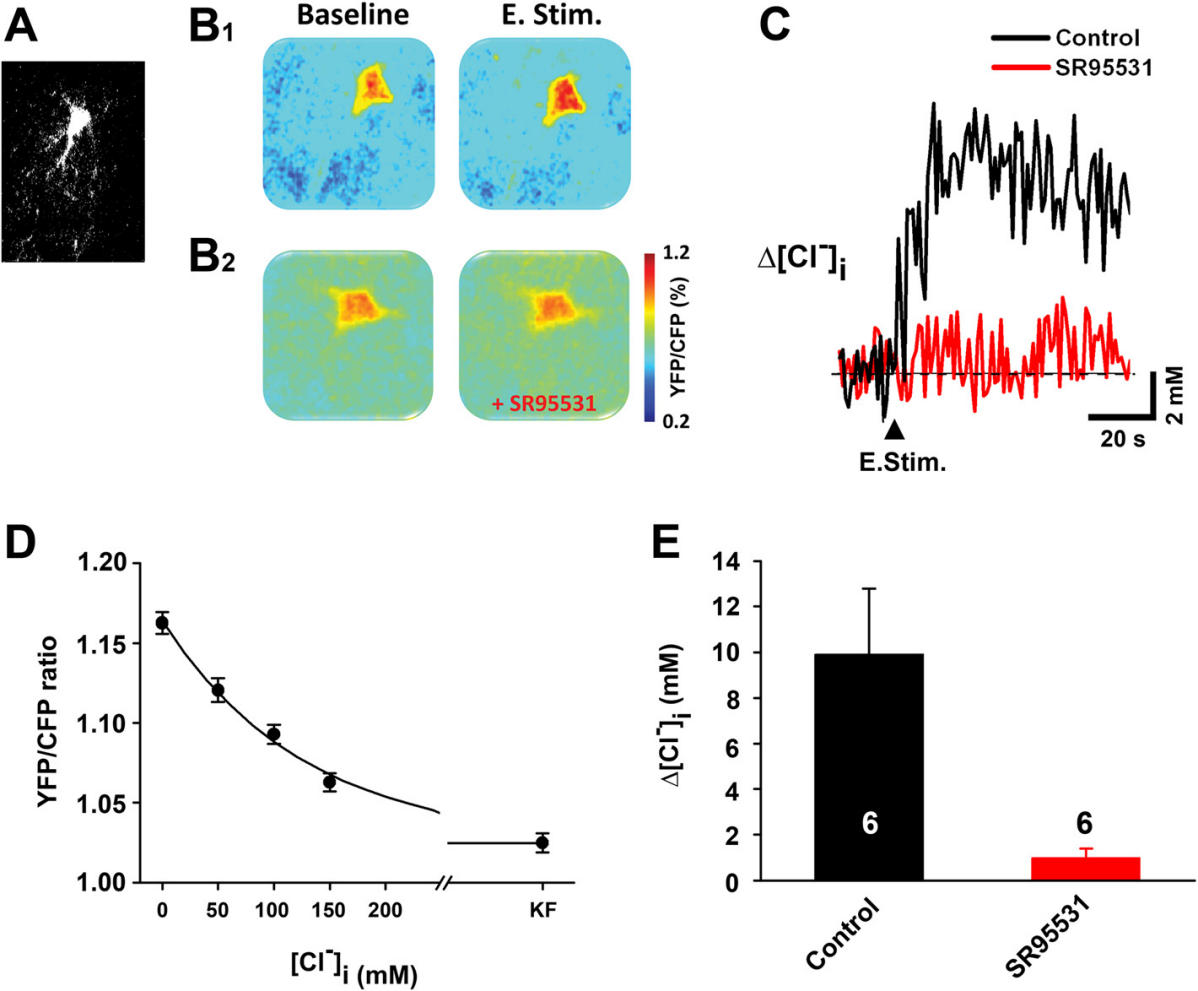
**Single-cell 2-photon Cl**^**-**^**imaging in Adeno-clomeleon-GFP virus infected mouse sensorimotor cortical slice.A**. Sample image of neuron with Adeno-clomeleon-GFP virus infection. **B1**: Two panels on right are color-coded images showing [Cl^-^]_i_ in an upper layer pyramidal neuron. Note that [Cl^-^]_i_ is greater in the image acquired after L6/WM stimulation (E. Stim.) than in image acquired before L6/WM stimulation (Baseline). Each color-coded image is average of 15 consecutively acquired images. **B2**: Same format and neuron as in B1, but in this case the images were obtained in the presence of GABA_A_ receptor antagonist SR95531 (gabazine). In the presence of gabazine, L6/WM stimulation was associated with little or no increase of this neuron’s [Cl^-^]_i_. **C**: Time course of alteration of neuronal [Cl^-^]_i_ (Δ[Cl^-^]_i_) associated with L6/WM stimulation. Data points were obtained from images of same neuron before (black plot) and after exposure to ACSF containing the GABA antagonist gabazine (red plot). Error bars indicate ±1SE. **D**: Calibration curve of Cl^-^ for FRET signals. Data points were obtained from standard solution containing 0, 50, 100, and 150 mM chloride respectively. **E**: Bar with black color (E. Stim.) shows average across-neuron [Cl^-^]_i_ (n = 6) measured subsequent to L6/WM stimulation; bar on right (E. Stim. + SR95531; red color, n = 6) shows average across-neuron [Cl^-^]_i_ measured subsequent to L6/WM stimulation in the presence of the GABA_A_ antagonist SR95531. All three bars are normalized by [Cl^-^]_i_ before stimulation. Each neuron was located in a different slice. ** indicates p < 0.001; * indicates p < 0.05; NS – not significant.

Slices of sensorimotor cortex from mice with Adeno-clomeleon-GFP virus infection (Figure 3A) were studied in an attempt to directly evaluate whether the L6/WM stimulus that was utilized (1 s duration, suprathreshold, 20 Hz) is, in fact, accompanied by a significant increase of [Cl^-^]_i_ in the upper layer pyramidal neurons located in the region of the slice immediately above the stimulus site. Sensorimotor cortex of the virus infected mouse was viewed as well suited for addressing this issue because it enables imaging of neuronal [Cl^-^]_i_ within the time scale and magnitude of the alterations required to satisfactorily account for our finding (see Figures 2) that GABA application evokes pyramidal neuron depolarization for ~15 s subsequent to a 1 s, 20 Hz suprathreshold L6/WM stimulus.

Experiments with this model/approach yielded observations demonstrating – using the CFP:YFP ratio as an indicator of the status of neuronal [Cl^-^]_i_ – that [Cl^-^]_i_ not only is increased in individual upper-layer pyramidal neurons following L6/WM stimulation (Figure 3B1), but the increase of neuronal [Cl^-^]_i_ that normally follows the L6/WM stimulus fails to occur when the ACSF used to bathe the slice contains 10 μM of gabazine (SR95531) – a highly selective GABA_A_ receptor blocker (Figure 3B2). Time course of [Cl^-^]_i_ change evoked by 1 s, 20 Hz stimulation was plotted in Figure 3C. The value from this imaging data were converted to chloride concentration as mM level with using calibration curve which was obtained from standard solution containing 5 different concentration of chloride ion (Figure 3D). The chloride ion in pyramidal neurons was increased about 9.92 ± 2.86 mM after 1 s 20 Hz L6/WM stimulation which was inhibited with gabazine application (0.98 ± 0.41 mM).

The effect of L6/WM stimulation on pyramidal neuron [Cl^-^]_i_ was evaluated in a total of 6 slices, each obtained from a different mouse subject. The imaging data generated by these experiments (summarized by the bar plot in Figure 3E) revealed that not only is the 1 s 20 Hz L6/WM stimulus accompanied by a statistically highly significant elevation of upper layer pyramidal neuron [Cl^-^]_i_, but exposure of the slice to ACSF containing 10 μM gabazine is associated with a statistically significant ***suppression of the increase of neuronal [Cl***^***-***^***]***_***i***_ which otherwise normally accompanies L6/WM stimulation. The authors regard both the post-L6/WM stimulus elevation of pyramidal neuron [Cl^-^]_i_ and its attenuation/elimination by gabazine as fully compatible with the proposal that an exposure to extrinsic excitatory afferent drive is accompanied by an elevation of the equilibrium potential of GABA_A_ channels in pyramidal neurons (due to increased neuronal [Cl^-^]_i_) and, as a consequence, the response of an upper layer sensorimotor cortical pyramidal neuron to GABA is depolarizing for ~15 s subsequent to such a L6/WM stimulus.

### L6/WM stimulation triggers a GABA-mediated increase of pyramidal neuron [Ca^2+^]_i_

**Figure 4:**
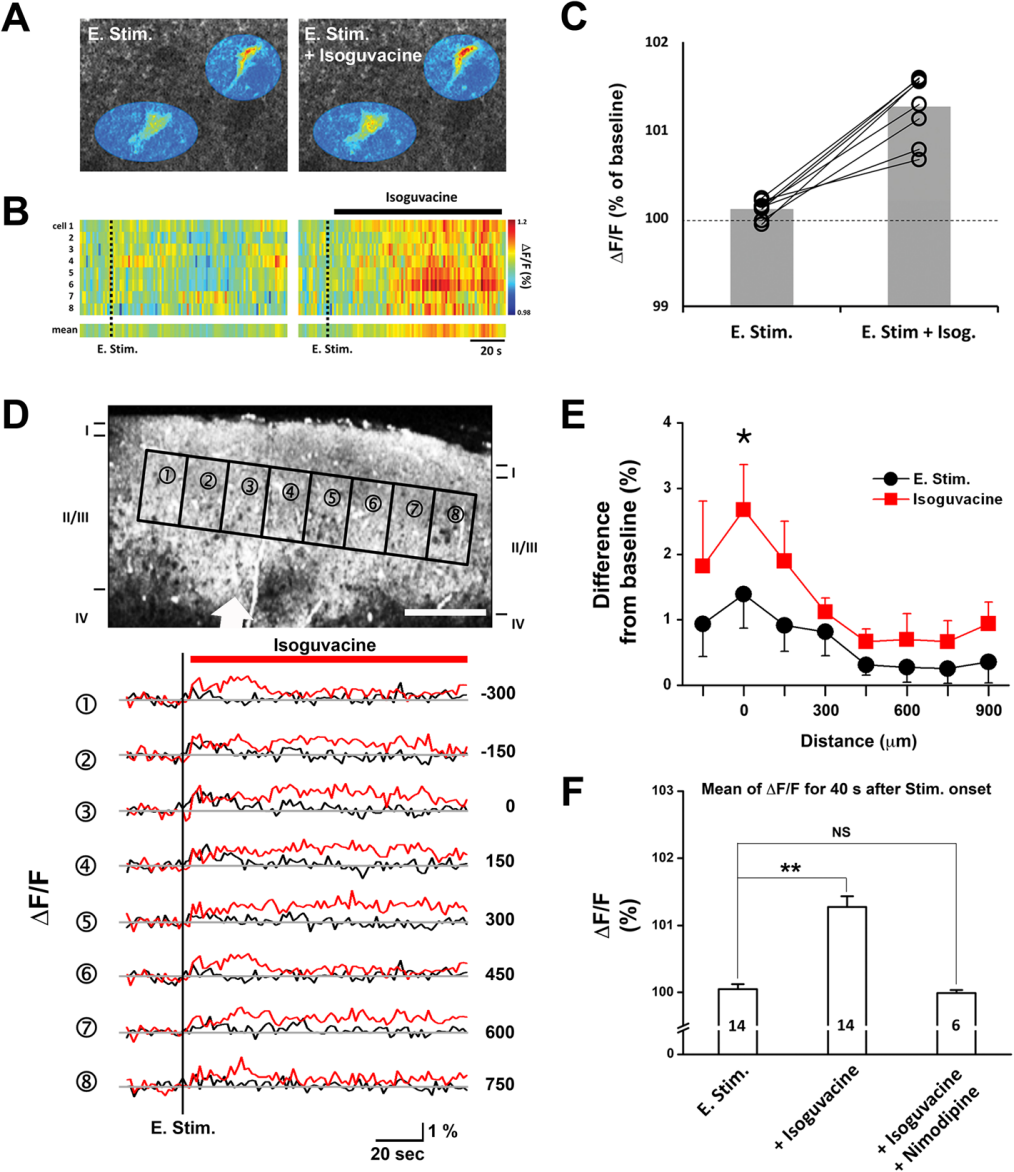
**Single-cell and population-scale 2-photon Ca**^**2+**^**imaging in Fura-2 AM stained cortical slices.A**: Two representative Fura-2 AM stained upper-layer pyramidal neurons imaged for 80 s starting 20 s after L6/WM electrical stimulation: (i) in normal ACSF (left panel); and (ii) in ACSF containing GABA_A_ agonist isoguvacine (right panel). **B**: Time-course of stimulation-induced increase of [Ca^2+^]_i_ for each of the 8 upper-layer pyramidal neurons imaged in normal ACSF (left), and in the presence of 10 μM isoguvacine (right). Vertical bar at right indicates color coding of normalized magnitude of stimulus-evoked increase of fluorescence (ΔF/F). **C**: Time-averaged post-stimulation increase of [Ca^2+^]_i_ for each of the 8 neurons which provided the data shown in B. Fura-2 AM signal was averaged over 40 s beginning 20 s after the end of stimulation. **D**: Representative image for indication of ROI (1 to 8) of measuring Ca^2+^ signal across layer 2–3 in cortical slice. White arrow: location of electrical stimulation. Stimulus induced individual trace of 1 to 8 ROI before (black traces) and with application of isoguvacine (red traces) was shown in lower panel of C. Numbers in right side indicates the distance (μm) from stimulation. **E**: Average across-slice (n = 4) Ca^2+^ signal in layers 2–3 evoked by L6/WM stimulation across 1.2 mm slice sector in the presence (gray squares) or absence (black circles) of isoguvacine. In each of the 4 slices, the 1.2 mm slice sector was divided into 8x250 μm rectangular compartments. Error bars = ±1SE. **F**: Bar plot summarizing the Ca^2+^ imaging observations obtained under the following conditions. Numbers in bars indicate number of observations obtained under each condition; ** indicates statistical significance of difference between bracketed values is <0.001; NS indicates difference between bracketed values is not significant.

The demonstration that for ~15 s following a period of vigorous stimulus-evoked activity the synaptic effect of GABA on upper layer pyramidal neurons is depolarizing made the authors view it as likely that the neurons experiencing this conversion should also exhibit increased [Ca^2+^_i_ (see [23], also [24], for review of evidence demonstrating the effect of GABAergic synaptic transmission on neuronal [Ca^2+^_i_). To evaluate this possibility slices of sensorimotor cortex (mouse) were stained with the Ca^2+^ indicator Fura-2 AM, and 2-photon microscopy at 20× magnifications was used to monitor pyramidal neuron Ca^2+^ (Figure 4A) in slices of sensorimotor cortex obtained from B6 mice.

Each of the color-coded images in Figure 4A shows the same two Fura2-AM stained upper layer pyramidal neurons. The neurons were imaged both prior to and following the application of a 1 s, 20 Hz suprathreshold electrical stimulus to a L6/WM site located directly below the cell column occupied by the neurons. Comparison of the left and right color-coded images in Figure 4A unambiguously shows (compare image labeled “E. Stim.” with image labeled “E. stim. + Isoguvacine”) not only that the L6/WM stimulus resulted in an above background Ca^2+^ signal when the slice was bathed by normal ACSF, but that this signal was enhanced substantially when the ACSF that perfused the slice contained 10 μM of the selective GABA_A_ agonist isoguvacine – i.e., a greater post-stimulation increase of [Ca^2+^]_i_ occurred in both neurons when the ACSF used to perfuse the slice contained the GABA agonist isoguvacine.

Figure 4B shows the time-course of the L6/WM stimulus-induced alteration of [Ca^2+^]_i_ detected in the 8 upper layer pyramidal neurons that we studied using the above-described approach (in 4 slices from 4 different mice). The data obtained from each of these 8 neurons are shown by a single color-coded, horizontal display/timeline; and the number at left of each line (#1-#8) identifies the neuron which provided the data. Ca^2+^ monitoring at single-cell resolution was achieved by integrating the signal over the neuronal soma in each image frame (images were acquired every 0.25 s). The raster-like display of the Ca^2+^ signal shown in the left panel of Figure 4B makes it visually apparent that in the absence of isoguvacine a transient (lasting <20s), but relatively minor increase of [Ca^2+^]_i_ occurred following the L6/WM stimulus in the majority (6/8) of the neurons. In contrast, when 10 μM isoguvacine was released into the recording chamber immediately subsequent to L6/WM stimulation, that stimulation was accompanied by a substantially larger and longer-lasting elevation of [Ca^2+^]_i_ in each of the 8 neurons (see the right panel in Figure 4B). The bar plot in Figure 4C summarizes the highly significant average across-neuron (n = 8) enhancement of the L6/WM-induced increment of neuronal [Ca^2+^]_i_ that occurred when the ACSF that bathed the slice contained isoguvacine.

Considered collectively, the findings are viewed as consistent with the idea that for ~15 s subsequent to L6/WM stimulation the effect of isoguvacine binding to the GABA_A_ receptors of pyramidal neurons in cell columns located above the L6/WM stimulus site is augmentation of the depolarization associated with L6/WM stimulation and, as a result, the neurons in this location in the sensorimotor cortical slice exhibit a larger-than-normal post-stimulation increase of [Ca^2+^]_i_.

Additional Ca^2+^ imaging experiments were carried out in an attempt to identify more precisely the dimensions of the region of the sensorimotor cortical slice in which the synaptic action of GABA is transformed from hyperpolarizing to depolarizing subsequent to L6/WM stimulation. To this end, layers 2–3 in Fura-2 AM stained slices again were studied using the 2-photon Ca^2+^ imaging method, but this time using wide-view microscopy (Figure 4D). The images obtained from the 1.2 mm sector of each slice evaluated using this approach were: (i) partitioned into 8, 150 μm-wide, box-shaped regions (ROI) that spanned layers 2–3, (ii) average Fura-2 AM intensity within each ROI was determined for each successive image frame acquired during the period between 20s before and 75 s subsequent to termination of the L6/WM stimulus, and (iii) the resulting Fura-2 AM intensity values for each ROI were plotted as a time series (Figure 4C, see bottom panel).

Comparison of the observations obtained from 4 slices both before and after each slice was exposed to isoguvacine (Figure 4E) reveals that although, as expected, the Ca^2+^ signal triggered by L6/WM stimulation was enhanced by the GABA agonist, the increase of the Ca^2+^ signal occurred only in a spatially restricted sector of the region of the slice. That is, isoguvacine’s enhancement of the Ca^2+^ signal evoked by L6/WM stimulation was “spatially selective” in that it occurred only within the 200-300 μm sector of layers 2–3 above the L6/WM stimulus site. Furthermore, when the ACSF used to perfuse the recording chamber not only contained the GABA agonist isoguvacine, but also included 2 mM nimodipine (an L-type Ca^2+^ channel blocker), no enhancement of the Ca^2+^ signal occurred following L6/WM stimulation (Figure 4F). This result is interpreted to indicate that the enhancement by isoguvacine of the Ca^2+^ signal triggered in upper layer pyramidal neurons by L6/WM stimulation is attributable to: (i)Isoguvacine binding to GABA_A_ receptors, (ii)Cl^-^ efflux from pyramidal neurons via GABA_A_ channels resulting in membrane depolarization which, in turn, leads to Ca^2+^ influx via L-type Ca^2+^ channels and increased [Ca^2+^]_i_ in the affected neurons.

## Discussion

### Overview of results

The effect of extracellularly applied GABA on upper layer pyramidal neurons in rodent sensorimotor cortical slice was evaluated: (i)In the absence of excitatory afferent drive, (ii)Subsequent to supra threshold repetitive electrical stimulation of a site at the junction between layer 6 and the underlying white matter. The major experimental findings and the authors’ view of their significance are as follows:

1. The synaptic action of GABA on layer 2–3 sensorimotor cortical pyramidal neurons at the center of the column-shaped cortical region responding to suprathreshold 1 s, 20 Hz electrical stimulation of a L6/WM site is bipolar (+/−) and activity-dependent. More specifically, during the ~15 s period subsequent to the L6/WM stimulation GABA produces neuron depolarization, whereas at rest (prior to stimulation or more than 15 s after stimulation) GABA produces hyperpolarization (Figures 2).

2. The post-stimulus increase of [Cl^-^]_i_ exhibited by pyramidal neurons at the center of the column-shaped cortical region responding to suprathreshold 1 s, 20 Hz electrical stimulation of a L6/WM site requires GABA binding to GABA_A_ receptors. This interpretation is based on the finding (Figure 3) that neurons in this sector exhibit little or no increase of [Cl^-^]_i_ in the presence of 10 μM gabazine – a selective GABA_A_ receptor antagonist.

3. Furthermore, the elevation of neuronal [Ca^2+^]_i_ that occurs following L6/WM stimulation, like the post-stimulus increase of neuronal [Cl^-^]_i_, appears attributable, in part, to GABA binding to GABA_A_ receptors because in the presence of isoguvacine – a selective GABA_A_ receptor agonist – this poststimulus increase of neuronal [Ca^2+^]_i_ is greatly enhanced (Figure 4).

### Relationship of findings to observations reported previously

Brief mechanical stimuli applied simultaneously at multiple skin sites not only result in a spatially funneled tactile percept in humans [25-27], but also in a spatially funneled response of the contralateral SI cortex in the cat [28] and nonhuman primates [29,30]. In addition to its demonstration using multi-site skin stimulation, tactile spatial funneling has also been successfully demonstrated in primate SI using repetitive single-site skin stimulation [31,32]. Because of the widespread distribution of axon projections from the somatosensory thalamus to SI [33-36] and the extensive spread of lateral excitatory connections within SI [37], stimulation of a single point on the skin evokes activity in a spatially extensive territory in SI [38,39]. When stimulation continues, however, this initially very extensive territory rapidly shrinks/funnels to an ~1 mm diameter cortical region [31,32,40]. Similarly in the rodent somatosensory cortical slice, tetanic L6/WM stimulation evokes activity initially in a large cortical territory, but that activity then quickly funnels into ~200-300 μm column [20-22,41].

It is established that GABAergic synaptic transmission mediated by GABA_A_ receptors is the main source of inhibition in cerebral cortex. While GABA_A_-mediated synaptic transmission is excitatory in the cortex of immature subjects, in adults it is inhibitory due to alteration of the neuronal transmembrane Cl^-^ concentration gradient brought about by developmental changes in the expression of anion co-transporters that result, in turn, in a lowering of the GABA_A_ reversal potential (E_GABA-A_) below the resting membrane potential [18,42]. Nevertheless, in adult neocortical neurons GABA action on GABA_A_ receptors has been shown to be excitatory in some circumstances. In particular, because of the uneven distribution of Cl^-^ ion channels E_GABAA_ is substantially higher in the dendrites of pyramidal neurons than in their somata, and as a result GABA release at the distal dendrite can be depolarizing [43]. In addition, the effect of GABA release by chandelier cells at their synapses on the initial axon segments of pyramidal cells can be excitatory/depolarizing due to the elevated presence of NKCC1, chloride co-transporter, in that location [9,44,45]. More directly relevant to the experimental findings presented in this paper, however, are studies which have convincingly shown that GABA released near somata of adult hippocampal pyramidal cells can become excitatory immediately following local tetanic stimulation, which produces a shift in neuronal [Cl^-^_i_ and an accompanying elevation of E_GABA_ (e.g., [7,8,10-13,17,42]. While such a stimulation-induced reversal of GABA effect has been most thoroughly studied in hyppocampal slices, Kaneda et al. (2005) [46] reported similar effects on neocortical neurons as well. Also, indirect evidence of a significant contribution of a switch of GABA’s synaptic action from hyperpolarizing/inhibitory to depolarizing/excitatory during normal somatosensory cortical functioning and perception derives from a recent [14] study of the response of neurons in area 3b of primate SI to vibrotactile skin stimulation in the presence of noxious skin stimulation. That study proposed that in SI cortical columns strongly activated by vibrotactile stimulation GABA action mediated via GABA_A_ receptors not only reliably becomes less inhibitory/suppressive, but can become depolarizing/excitatory.

### Impact of the spatially-selective action of GABA on the slice’s response to L6/WM stimulation

The observations in Figure 4 are viewed as consistent with the following functionally intriguing possibility: i.e.*,* because of its prominent activity-dependence, the response of a pyramidal neuron in the slice to GABA depends on its position relative to the L6/WM stimulus. More specifically, this idea proposes that: (i) GABA triggers maximal neuronal depolarization ***only*** within the 200-300 μm wide, column-shaped region which lies immediately above the L6/WM stimulus site; (ii)neurons in cell columns located at progressively larger radial distances from the above-described 200-300 μm region undergo progressively less GABA-evoked depolarization until a location in the slice is reached (the “null point”) where GABA action on GABA_A_ receptors has no impact on neuronal membrane potential; and (iii)for neurons in cell columns located at even greater distances from the L6/WM stimulation site the effect of GABA becomes increasingly hyperpolarizing until a point is reached at which it re-attains the magnitude of its hyperpolarizing effect on neurons “at rest”.

### Implication of the role in tactile perception

As a consequence of (i) the spatial selectivity of GABA’s action on GABA_A_ receptors, and (ii) the time required for development of the cellular-level events which underlie the alteration of the sign (+/−) of GABA action on the GABA_A_ receptors of pyramidal neurons (e.g., the gradual elevation of neuronal [Cl^-^]_i_ that occurs during an exposure to vigorous excitatory afferent drive), the spatiointensive profile of the response of the slice is viewed by the authors to undergo dynamic, but systematic funneling modification during the 1 s exposure to L6/WM stimulation. As a result, the slice gradually develops a response which possesses substantially greater spatial contrast than exists in the pattern of excitatory afferent drive evoked by the L6/WM stimulus, thereby more accurately representing/encoding the site of skin stimulation.

If it turns out that the bipolar, activity-dependent character of GABA’s action on pyramidal neurons in the rodent sensorimotor cortical slice is applicable to GABA’s role in stimulus information coding/representation by primary somatosensory cortex in an intact subject (for experimental observations regarded as consistent with this possibility see [22,31,32,39,41,47]), it is anticipated that the contributions of a bipolar, activity-dependent action of GABA to tactile spatial discriminative capacity would be substantial. The possibility that bipolar, activitydependent GABA action contributes to SI stimulus information encoding/representation and tactile discriminative capacity merits critical experimental evaluation because the results not only advance the relatively limited current understanding of the role of GABA in somatosensory cortical information processing, but enable appreciation for the first time of the role of activitydependent, bipolar GABA action in the rapid alterations of primary sensory cortical neuron stimulus feature selectivity believed to underlie perceptual learning (for status of the literature relevant to this issue see [2,48-55]).

## Methods

All experiments were performed in accordance with National Institutes of Health guidelines, and utilized protocols approved by the University of North Carolina Animal Care Committee.

### Cortical slice preparation

Sensorimotor cortical slices (thickness 350-400 μm) were obtained either from young (age 3–4 or 7–8 weeks) adult rats (Sprague–Dawley, Charles River) or C57BL/6 mice. Following decapitation the brain was rapidly removed and placed in cold artificial cerebrospinal fluid (ACSF) having the following composition (in mM): 118 NaCl, 4.8 KCl, 2.5 CaCl2, 25 NaHCO3, 1.2 MgSO4, 1.2 KH2PO4, 10 glucose. The slices were made using an oscillating tissue slicer (Vibratome 3000) at 3°C and stored in a bath of warmed (30°C), oxygenated (95% O2, 5% CO2) ACSF for a recovery period of at least 1 h. Each slice that was studied was transferred from a recovery/holding reservoir to the recording chamber of a fixed-stage upright microscope (Olympus BX51WI) and submerged in the 28–30°C oxygenated ACSF that was supplied to the chamber at a rate of 1.5–2 ml/min. The composition of the ACSF used to perfuse the recording chamber was the same as that in the recovery chamber. The submerged slice was visualized either directly via the microscope’s optics, or indirectly via a high resolution CCD camera system (CCD-100, Dage-MTI, Inc) that received the output of a CCD camera attached to the microscope’s video port.

### Optical intrinsic signal (OIS) imaging

The *in vitro* OIS imaging methods that were employed have been described in detail in previous publications [20,21,56]. Briefly summarized, each slice was submerged and positioned in the recording chamber of the stereomicroscope, trans-illuminated using white light from a controlled light source, and viewed from below at low-power magnification (×4). A bipolar tungsten electrode (FHC, outer diameter - 250 μm) was placed into contact with the L6/WM junction at a central location in the slice. Each exposure to electrical stimulation: (i) was 1 s in duration; (ii) consisted of 0.2 ms square-wave, constant-current pulses delivered at 20 Hz by a stimulusisolation unit attached to a programmable pulse generator (Master 8, AMPI); and (iii) was accompanied by the acquisition of a series of OIS images (acquisition rate – 4 images/s).

The initial image in the series was acquired 2 s prior to the first stimulus pulse. The OIS response of the slice to electrical stimulation at the L6/WM site was recorded using a cooled slow-scan CCD camera (Photometrics). Eight images acquired in association with each 1 s, 20 Hz exposure to suprathreshold L6/WM electrical stimulation were collected prior to stimulus onset, and the average of those 8 pre-stimulus images was used as the “no-stimulus reference” image for that series. The OIS difference images acquired subsequent to the onset of each L6/WM stimulation (“post-stimulus onset” images) were obtained by subtracting the no-stimulus reference image from each image acquired during this time period.

### Electrophysiological recording methods

#### General information

Whole-cell patch clamp recordings were obtained using borosilicate glass pipettes (resistance 4–6 MΩ) prepared using a 2-stage vertical pipette puller (Narishige PC-10). The pipettes were filled with a solution having the following composition (in mM): 120.0 Kgluconate, 20.0 KCl, 2.0 NaCl2, 20.0 HEPES, 0.5 EGTA, 10.0 glucose, 2.0 Na-ATP, 0.5 NaGTP; pH was adjusted to 7.3 with KOH. Patch recordings were obtained from pyramidal neurons under the same conditions (temperature, perfusion rate, etc.) used to obtain OIS imaging observations. Voltage-clamp and current-clamp recordings were acquired using a MultiClamp 700B amplifier, using pClamp9 software (Molecular Devices, Union City, CA, USA).

All patch recordings were from layer 2–3 pyramidal neurons located within the columnshaped cortical region that exhibited maximal OIS response to the L6/WM stimulus (Figure 1). All recordings were subjected to 50–500 Hz filtering. No correction for liquid junction potential was made.

### Viral injection

C57BL/6 mice (7–8 weeks old) were anesthetized by intraperitoneal injection of by 2% avertin (20 μl/g) and were placed in stereotaxic frame. Adeno-clomeleon-GFP virus was loaded in a microdispenser and injected into the somatosensory cortex at a rate of 0.2 μl/min (total 2 μl) using a syringe pump with a 25 μl syringe. The stereotaxic coordinates of the injection site were following as anteroposterior, −1.7 mm; lateromedial, ±1.7 mm; dorsoventral, −1.50 mm from bregma.

### Two-photon Cl^-^ and Ca^2+^ imaging

A 2-photon microscopy system (TrimScope, LaVisionBioTec, Bielefeld, Germany) was used to: (i) assess neuronal [Cl^-^_i_ in slices of sensorimotor cortex from measurement of FRET-dependent emission account for change of [Cl^-^_i_[57,58] and (ii) alterations of neuronal [Ca^2+^_i_ in sensorimotor cortical slices from B6 mice. An upright microscope (Olympus BX51 WI, Japan) equipped with a water immersion lens (0.95 W, XLUMPlanFI, Olympus, Germany) enabled ratiometric Cl^-^ and Ca^2+^ imaging. The excitation source was a mode-locked Ti/Sapphire laser (Mai Tai; Spectra Physics, Santa Clara, CA) tuned to 800 nm. The imaging software that was utilized (Inspector Pro; LaVisionBioTec, Bielefeld, Germany) allows simultaneous recordings of two fluorescent signals (CFP filter: 500 SP; YFP filter: 535 BP 50) as fluorescence resonance energy transfer (FRET) signals. Time series of images were acquired from clomeleon-expressing cells using a range of image geometries (64 × 8 to 256 × 256 pixels) with a scan rate of 2 ms/line. The images then were analyzed offline with MetaMorph version 7.6 (Moleculardevices, Sunnyvale, CA) and Matlab software. In the Ca^2+^ imaging experiments the slice was transferred to the recording chamber of the microscope after a 30 min period of incubation in ACSF containing 5 μmol/L Fura-2 AM and 1 μmol/L pluronic acid (Molecular Probes) at room temperature. External solution for dye staining contained (in mM/L) 150 NaCl, 10 HEPES, 3 KCl, 2 CaCl_2_, 1 MgCl_2_, 1 sucrose, and 10 glucose (pH was adjusted to 7.4 and osmolarity to 325 mOsm). The laser was tuned to 810 nm. Recordings of signal intensity and conversion to [Ca^2+^_i_ were performed using MetaMorph version 7.6 (Molecular Devices) and Matlab software.

## Competing interests

The authors declare that they have no competing interest.

## Authors’ contributions

JL, JW, OF, MT, CJL and BW conceived of the project and designed experiments. JL and JW performed all experiments, analyzed data. JL, CJL, and BW wrote the manuscript. All authors read and approved the manuscript.
